# Patient and visitor aggression de-escalation training for nurses in a teaching hospital in Cairo, Egypt

**DOI:** 10.1186/s12912-022-00828-y

**Published:** 2022-03-18

**Authors:** Dena Ali Abozaid, Mohamed Momen, Nahla Fawzy Abou El Ezz, Hanaa Abdelhakiem Ahmed, Mahi Mahmoud Al-Tehewy, Maged El-Setouhy, Mohamed El-Shinawi, Jon Mark Hirshon, Moustafa El Houssinie

**Affiliations:** 1 Community, Environmental and Occupational Medicine Department, Faculty of Medicine, Ain Shams University, a national university in Egypt, Cairo, Egypt; 2 Community Health Department, Faculty of Nursing, Ain Shams University, Cairo, Egypt; 3 Department of Family and Community Medicine, Faculty of Medicine, Jazan University, Jazan, Kingdom of Saudi Arabia; 4 Department of Surgery, Faculty of Medicine, Ain Shams University, Cairo, Egypt; 5 Vice president, Galala University, Suez, Egypt; 6 Department of Emergency Medicine, University of Maryland School of Medicine, Baltimore, MD USA

**Keywords:** Occupational Violence, Aggression, Healthcare, Hospitals, Training

## Abstract

**Background:**

Workplace violence (WPV) has been recognized as a major occupational hazard worldwide. Healthcare professions are particularly at a higher risk of WPV. Patients and their relatives are commonly the most common perpetrators for WPV against physicians. Trainings on the universal precautions of violence, how to effectively anticipate, recognize and manage potentially violent situation is recommended by OSHA as a part of a written, effective, comprehensive, and interactive WPV prevention program.

**Objective:**

To implement and evaluate the effectiveness of a training session delivered to nurses. The training session aimed to increase nurses’ ability to identify potentially violent situations and to effectively manage these situations in a teaching hospital in Egypt.

**Methodology:**

A total of 99 nurses attended the training sessions. Confidence in coping with aggressive patient scale, along with nurses’ attitudes toward WPV, were used to assess the effectiveness of the training sessions.

**Results:**

Nurses’ perceived confidence to deal with aggression increased after attending the training sessions. Nurses’ attitudes toward WPV positively changed after attending the training session.

**Conclusion and recommendations:**

Increasing awareness of the problem among healthcare professions as well as the public is warranted. Violence prevention program with a zero-tolerance policy is warranted.

## Introduction

Violence in the workplace is the act or threat of violence directed toward persons at work or on duty, ranging from verbal abuse to physical violence [1]. WPV in the healthcare sector is a growing problem worldwide [2].

In 2002, a joint program conducted to investigate WPV against HCPs specifically in hospital wards, aiming to develop a framework for managing WPV in healthcare sector. In the program 2014 report, it stated that, through country case surveys in Thailand, South Africa, Portugal, Bulgaria, Brazil, and Lebanon, more than halfA of surveyed HCPs had experienced at least one incident of violence in the previous year, and that, there were no specific policies to prevent, nor to respond to WPV. This contributes to under-reporting of WPV incidents [3].

Previous literature showed that, WPV is a prevalent problem among HCPs worldwide, developed as well as developing countries [4–7]. Previous literature showed that, WPV is a prevalent problem among HCPs in Arab countries as well [8–12]. In Egypt, previous studies showed that WPV is a major problem against HCPs in hospitals [13–17]. As well as among nursing students as well [18]. Visitors, as well as patients themselves were the most common perpetrators of on job violence against HCPs [4, 9, 12, 15–17]. Miscommunication between patient and doctor are commonly the cause of escalating into violent incidents [19]. Moreover, only half of on job violence victims reported the incident. Half of the participants described reporting as ineffective, two thirds of the participants were even aware that a WPV reporting system existed in ASUHs [15].

HCPs exposed to WPV suffer from emotional consequences. Victims of WPV report feeling hurt, feared, ashamed, angry, powerless, shock, embarrassed and intimidated. These emotional consequences lead to re-victimization and negative self-evaluation [10, 20]. WPV is considered an important cause of post-traumatic stress disorder among HCPs. Up to 70% of WPV victims reported symptoms of PTSD [3]. Not only has WPV against HCPs, negative impact on psychological and physical well-being of HCPs, but also affects their job motivation, consequently compromising quality of healthcare, and putting healthcare provision at risk. WPV also leads to enormous financial loss in the health sector [21]. Nurses who experienced WPV were more likely to consider quitting their jobs (OR = 3.9; CI 1.8–8.3) [10].

The potential for WPV can be minimized if effective prevention procedures are adopted within the organization. The threat of WPV can be reduced if potentially aggressive behavior is reported and/ or identified as early as possible, and effectively dealt with [22].

Patients’ violence against HCPs is usually recognized as unintentional, which may contribute to underreporting and even acceptance of WPV [23].

Previous literature shows that WPV against HCPs is a prevalent problem. Visitors, as well as patients themselves were the most common perpetrators [9].

Hospitals are perceived as a stressful environment by patients and visitors. This leads patients to react aggressively. understanding trauma, neuroscience of threat and safety can help HCPs in understanding patients’ aggression and develop strategies to better cope with it [24]. Breaking bad news is a common volatile situation that is potentially violence and require certain skills and approach to communicate and prevent its escalation into violence against HCPs [25].

Trainings on the universal precautions of violence, how to effectively anticipate, recognize and manage potentially violent situation is recommended by OSHA as a part of a written, effective, comprehensive, and interactive WPV prevention program [26]. Previous studies assessed the effectiveness of aggression de-escalation training program and reported a statistically significate difference in participants confidence to deal with aggression after attending the training program [27, 28].

Giving the importance of WPV in healthcare sector and its consequences, not only on the HCPs, but also on the quality of health services [21]. This study aimed to implement and study the effectiveness of a training session delivered to nurses. The training session aimed to increase nurses’ ability to identify potentially violent situations and to effectively manage these situations in a teaching hospital in Egypt.

## Subjects and methods

### Study design

An Intervention Study (One-Group Quasi-Experimental Longitudinal Pretest/Posttest Design).

### Study settings

The study was conducted in Ain Shams University Hospitals (ASUHs), one of the largest tertiary teaching institutions in Cairo, which provide health services to patient from all over Egypt, with an average of 775,000 medical visits annually 2300 medical bed [29].

### Study population and recruitment

Nurses attending in the wards in all main hospitals in ASUHs which specialized in internal medicine, surgery, pediatric, Gynecology and Obstetrics, Cardiothoracic surgery hospital and Oncology department Hospital. All nurses working in the wards in in ASUHs, agreed to participate in the study were eligible. Recruited nurses attended a training on workplace violence aiming to empower them with skills needed to effectively identify and manage workplace violence.

The training was proposed to the ASUHs administration. Announcements of the training session dates were sent ahead to nurses working in wards in all departments in ASUHs. Training sessions’ attendance was organized internally in the wards according to the workload in coordination with the training and quality units in ASUHs. A total of 10 training sessions were conducted from January 2018, till August 2018.

### Sampling method

A Convenience non-random sampling method was used.

### Sample size

The sample size was calculated based on the effect size concept, in our case it would be the paired difference in (Confidence in Coping with Patient Aggression CCPA scale), divided by the standard deviation of the difference. With a small to medium effect size of 0.4, level of significance of 0.05, power of 0.80 and using two sided Wilcoxon signed rank test [27], a sample size of 60 nurses was satisfactory. The calculations were done using G*Power 3.1.9.2 software.

Of all nurses who attended the training sessions, a total of 99 nurses agreed to participate in the study and completed the pretest/posttest surveys, only 65 of them were reachable and completed a one-month follow up survey.

### The intervention

#### Training curriculum: universal precautions for WPV in healthcare sector [26, 30, 31]

An evidence based two-hour training session for nurses working in ASUHs was tailored and conducted. An occupational medicine specialist and a psychiatrist specialist extensively reviewed and revised the training material, to ensure that the training materials met the requirements.

The training session aimed to 1- Improve nurses’ ability to identify potentially violent situations, 2- Provide nurses with the needed skills to effectively de-escalate a violent situation. The training was a multimedia two-hour session attended once. The training session included the following:

- Introduction: definition of WPV, types of WPV and magnitude of WPV in healthcare sector worldwide.
- Components of an effective and comprehensive violence prevention program and the importance of training.
- OSHA Universal precautions for job violence against HCPs: that the assault is usually Predictable and Preventable, and that it is important to recognize escalation and intervene immediately.
- What motivates the attacks? Causes of patient/visitor violence against HCPs in hospitals.
- Warning symptoms that the situation is potentially violent.
- Techniques to de-escalate a potentially violent situation: Resolution Strategies for Aggressive Patients

### Study tools

Evaluating the skill-based outcomes that the nurses would acquire from attending the training sessions was used to evaluate the effectiveness of the training session. The following surveys were used:

I. **Confidence in Coping with Patient Aggression scale (CCPA scale) **[32]**:** CCPA scale is a one-direction construct scale. It consists of 10-items, which reflects self-confidence to deal with physical and psychological aggression, where higher scores indicate more confidence in managing aggressive behavior.
II. **Nurses’ Attitudes toward violence\aggressive behavior questionnaire **[33, 34]**:** a questionnaire of nurses’ attitudes toward WPV was used to measure the effectiveness of the training session. The questionnaire consisted of 7 statements with a five-point Likert scale response. Higher scores indicated favorable attitudes. Nurses’ responses in the 7 attitudes statement were summed into a total attitude score.

CCPA scale and Nurses’ attitudes toward violence questionnaire were a self-administered surveys and were measured 3 times: a baseline at the beginning of the training session (pretest), immediately at the end of the training (posttest), and a one-month after attending the training session (one month follow up). Nurses’ responses were calculated and individual matching and comparisons between pretest, posttest and follow up was done.

### Tools validity

Both surveys were originally in English language and were translated into Arabic language and back translated into English language by an external reviewer. A comparison of the two languages versions was done, to confirm accuracy of the translation process and validity of the scale.

The Original CCPA scale showed a high degree of internal consistency and precision (Cronbach’s alpha = 0.92), reflecting the inter-correlation between CCPA scale items [13]. Cronbach’s alpha was measured based on nurses’ responses in our study, and it showed, similar to the original scale, a high degree of internal consistency and precision (Cronbach’s alpha = 0.88).

### Data management and analysis

The collected data was revised, coded, tabulated, and analyzed using Statistical package for Social Science (SPSS 25 for windows). ANOVA was used to compare individual nurses’ response between baseline, immediately after training, and follow up (one-month). Post hock test was used to identify the pairwise difference. Reported *p*-values were adjusted “Bonferroni adjustment” was applied. *P* value less than 0.05 was considered statistically significant. Comparison of nurses’ responses in attitudes toward violence between baseline, immediately after training and follow up (one-month) were analyzed as; 1- Quantitative scores using repeated measures one-way ANOVA, using post hock test to identify the pairwise difference. 2- Qualitative ordinal variable using test of marginal homogeneity to compare between paired scores. Bonferroni Adjustment for multiple comparisons was applied on reported results, there for results were considered statistically significant at a *p* value < 0.017. Both methods yield the same results. To avoid repetition, only analysis using test of marginal homogeneity is displayed.

## Results

A total of 99 nurses completed the pretest and posttest. Only 65 of them were reachable for a follow up and completed a one-month follow up survey. Nurses mean age was 39.9 ± 8.2 year. Most of nurses attended the training session were female (93.9%). Most of nurses were married (80.8%), only 10.1% were singles. There was no statistically significant difference in confidence in coping with aggressive behavior, and attitude toward aggression score, regarding age, years of experience, nor gender.

There was a significant effect of attending the training session on nurses’ perceived confidence with dealing with aggressive patients (*F* = 90.01, *p* < 0.001), with medium effect size (partial eta squared = 0.58). Bonferroni post hoc test showed that, nurses attended the training course had a higher posttest and one-month follow up scores, compared to their pretest score (Mean ± SD: 70.4 ± 22.7, 68.1 ± 16.7 and 39.8 ± 20.7 respectively). Differences between pretest and posttest, and between pretest and one- month follow up were statistically significant (*p* < 0.001 and *p* < 0.001) respectively. There was a statistically significant effect of attending the training session on the nurses’ “Attitudes toward violence / aggressive behavior questionnaire score” (*F* = 49.62, *p* < 0.001), with a moderate effect size (partial eta squared = 0.437). Bonferroni post hoc test showed that, nurses attended the training course had a higher posttest and one-month follow up scores, compared to their pretest score (Mean ± SD: 31.1 ± 3.4, 31.5 ± 2.9 and 27.0 ± 3.2 respectively). Differences between pretest and posttest, and between pretest and one- month follow up were statistically significant (*p* < 0.001 and *p* < 0.001) respectively.

Nurses’ posttest and one-month follow up scores in “comfortability for working with an aggressive patient” were higher, compared to their pretest score, difference between pretest/posttest, and between pretest/one- month follow up were statistically significant (*p* < 0.001 and *p* = 0.001) respectively (Table 1).

**Table 1:**
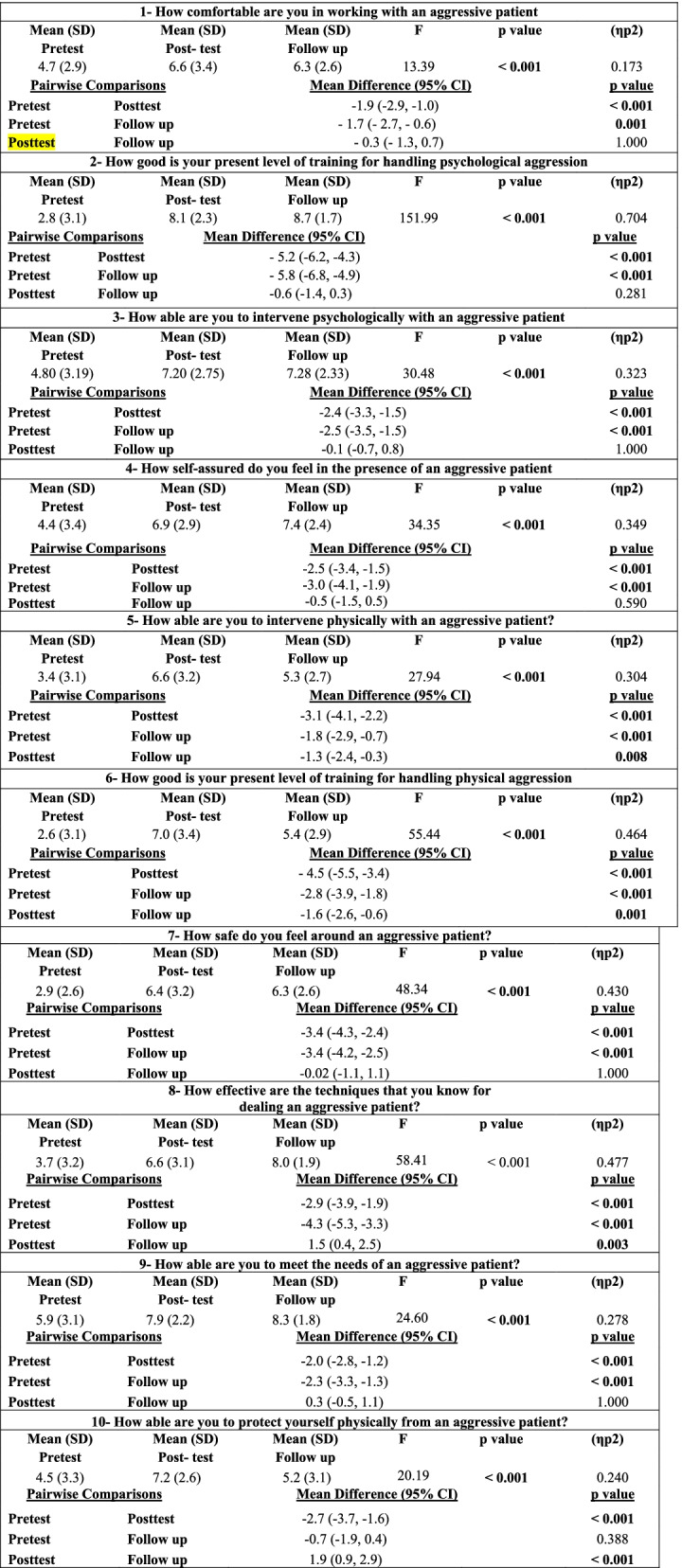
Nurses’ perceived confidence in coping with aggressive behavior scale items (*n* = 65)

Adjustment for multiple comparisons: Bonferroni, p significant at 0.05 level. Abbreviations: *SD* Standard Deviation, One-way repeated measured ANOVA (Analysis of Variance, *CI* Confidence Interval, (ηp2): partial eta squared.

One fourth of nurses strongly agreed that violence can be anticipated and recognized in pretest, compared to 47.5% of nurses in posttest, and 61.5% in follow up, pretest/posttest and pretest/follow up differences were statistically significant (*p* < 0.001, *p* < 0.001 respectively).

One third of nurses disagreed in pretest that “physical violence is part of the job”, compared to 51.5% and 70.8% of nurses immediately posttest and one-month follow up respectively. Test of marginal homogeneity was statistically significant for pretest/posttest and pretest/follow up (*p* < 0.001, *p* < 0.001 respectively) (Table 2)*.*

**Table 2:** Nurses’ Attitudes toward violence \ aggressive behavior questionnaire items

	**Pretest**		**Posttest**	**Follow up**		**Pair Comparison**	*p* Value*
Patients strike out because they are afraid							
SA	16 (16.2)		44 (44.4)	22 (33.8)		**Pretest/Posttest**	** < 0.001**
A	31 (31.3)		35 (35.4)	25 (38.5)		**Pretest/Follow up**	**0.001**
N	28 (28.3)		9 (9.1)	10 (15.4)		**Posttest/Follow up**	0.035
D	18 (18.2)		9 (9.1)	8 (12.3)			
SD	6 (6.1)		2 (2.0)	0			
Much of the aggression and violence I see at work is preventable							
SA	19 (19.2)		44 (44.4)	34 (52.3)		**Pretest/Posttest**	** < 0.001**
A	43 (43.4)		39 (39.4)	26 (40.0)		**Pretest/Follow up**	** < 0.001**
N	22 (22.2)		14 (14.1)	3 (4.6)		**Posttest/Follow up**	0.819
D	12 (12.1)		2 (2)	1 (1.5)			
SD	3 (3)		0	1 (1.5)			
Someone who is good at recognizing the signs can tell when a patient is becoming agitated							
SA	24 (24.2)		47 (47.5)	40 (61.5)		**Pretest/Posttest**	** < 0.001**
A	52 (52.5)		39 (39.4)	24 (36.9)		**Pretest/Follow up**	** < 0.001**
N	18 (18.2)		12 (12.1)	1 (1.5)		**Posttest/Follow up**	0.123
D	4 (4.0)		0	0			
SD	1 (1.0)		1	0			
Staff should be educated about the prevention and management of aggressive behavior as part of their ln-service education							
SA	58 (58.6)		72 (72.2)	54 (83.1)		**Pretest/Posttest**	**0.003**
A	29 (29.3)		23 (23.2)	10 (15.4)		**Pretest/Follow up**	**0.001**
N	11 (11.1)		4 (4.0)	1 (1.5)		**Posttest/Follow up**	0.827
D	1		0	0			
SD	0		0	0			
It is usually better to intervene sooner rather than later in aggressive situations							
SA	52 (52.5)		71 (71.7)	52 (80.0)		**Pretest/Posttest**	** < 0.001**
A	32 (32.2)		24 (24.2)	11 (16.9)		**Pretest/Follow up**	** < 0.001**
N	9 (9.1)		4 (4.0)	1 (1.5)		**Posttest/Follow up**	0.739
D	4 (4.0)		0	1 (1.5)			
SD	2 (2.0)		0	0			
Being verbally abused is all part of the job							
SA		17 (17.2)	5 (5.1)		1 (1.5)	**Pretest/Posttest**	** < 0.001**
A		17 (17.2)	6 (6.1)		2 (3.1)	**Pretest/Follow up**	** < 0.001**
N		8 (8.1)	8 (8.1)		9 (13.8)	**Posttest/Follow up**	0.069
D		29 (29.3)	35 (35.4)		12 (18.5)		
SD		28 (28.3)	45 (45.5)		41 (63.1)		
Being physically assaulted is all part of the job							
SA		11 (11.1)	2 (2.0)		0	**Pretest/Posttest**	** < 0.001**
A		11 (11.1)	3 (3.0)		1 (1.5)	**Pretest/Follow up**	** < 0.001**
N		10 (10.1)	4 (4.0)		4 (6.2)	**Posttest/Follow up**	0.043
D		33 (33.3)	39 (39.4)		14 (21.5)		
SD		34 (34.3)	51 (51.5)		46 (70.8)		

*Abbreviation: SA* Strongly Agree, *A* Agree, *N* Neutral, *D* Disagree, *SD* Strongly Disagree

*Pretest n* = *99, Posttest n* = *99, Follow up n* = 65. Percentages rounded to one decimal place *Pairwise comparison using test of marginal homogeneity, Bonferroni Adjustment for multiple comparisons, p significant at 0.017 level

## Discussion

The current study aimed to implement and study the effectiveness of an evidence-based training for nurses designed to improve their ability to identify patient/visitor violence, to effectively manage and to de-escalate potentially violent situations in ASUHs. To our knowledge, the current study is the first study of its kind in Egypt. Breaking bad news is a common volatile situation that is potentially violence and require certain skills and approach to communicate and prevent its escalation into violence against HCPs [25]. This highlights the importance of conducting this training.

Our study results are consistent with the findings of previous studies, which conducted trainings to provide nurses with the skills needed to de-escalate aggressive patients. Previous studies reported a statistically significant difference in the attendees’ CCPA scale total score between the baseline and the immediate posttest scores [27, 28, 35–37]. It was noted that none of the previous studies reported the effect size of attending the program [27, 28, 35, 36].

It was noted that, our participants showed low pertest confidence scores (Mean ± SD: 39.8 ± 20.7) compared to Story et al., where participant nurses scored higher in pretest (Mean ± SD: 55.1 ± 5.18). Post-test scores were more similar (Mean ± SD: 75.8 ± 4.56) in Story et al. study compared to (Mean ± SD: 70.4 ± 22.7 and 68.1 ± 16.7 post-test and follow up respectively in our study. This difference highlights the importance of conducting trainings to improve HCPs ability to manage patient/visitor violence in Egypt [37].

Nurses retained their confidence to deal with aggression after attending the training course in follow up assessment, which was one month after attending the training session. Consistent with Nau et al., 2009, in which follow up assessment was after 2 weeks of nursing students’ practical placement, which was nearly 4–8 weeks after attending the training program [27].

A study explored gender differences in aggression perception among of medical students, after attending a training on violence management, found that men initially perceived aggression as a less destructive, yet attending the training diminished the difference in aggression perception between male and females [38].

There was a positive change in nurses’ belief that “it is better to intervene early in de-escalating an aggressive patient after attending the training session” (test of marginal homogeneity, *p* < 0.001 for both pretest/ posttest and pretest/follow up). Consistent with Beech, 2001, who reported a statistically significant positive difference in nurses’ attitude after attending the training session (*p* = 0.019).

One of the objectives of the training session was to emphasis that, although it is important for HCPs to acquire skills needed to de-escalate aggressive situations, yet patient/visitor aggression against HCPs is not accepted, and it is not part of the job. Nurses’ attitudes changed positively after attending the session; nurses became more assertive that verbal abuse is not accepted in the workplace (test of marginal homogeneity, *p* < 0.001 for both pretest/ posttest and pretest/follow up). Nurses became more assertive that physical violence in the workplace is not accepted (Test of marginal homogeneity, *p* < 0.001, *p* < 0.001 and *p* = 0.043 for pretest/ posttest, pretest/follow up, posttest/follow up respectively). Beech, 2001, did not find a statistically significant difference in nurses’ attitude toward accepting WPV after attending the training [34].

### Limitations

Reported effectiveness of the training is based on a self-perceived change in nurses’ confidence. Comparing reported incidence of WPV before and after implementing the training was not applicable, as WPV is usually underreported.

## Conclusion

The study found that, attending a training session on aggression management, increased nurses’ perceived confidence to deal with aggressive situation, and positively influenced nurses’ attitude toward WPV in healthcare sector.

### Recommendations

This study showed that communication skills training is important in managing and diffusing a potentially violent situation. More care showed be given to communication skills curriculum and the universal precautions for violence, in medical education, as well as a continuous on job training is warranted. Violence prevention training is needed combined with a comprehensive violence management policy, combined with engineering measures and administration commitment, to prevent WPV in healthcare sector. More studies on evaluating the effect of HCPs’ mental and emotional well‐being on WPV is warranted.

## Data Availability

The datasets generated and analyzed during the current study are not publicly available due privacy reasons. But are available from the corresponding author on reasonable request.
